# Muscle Atrophy Induced by Mechanical Unloading: Mechanisms and Potential Countermeasures

**DOI:** 10.3389/fphys.2018.00235

**Published:** 2018-03-20

**Authors:** Yunfang Gao, Yasir Arfat, Huiping Wang, Nandu Goswami

**Affiliations:** ^1^Key Laboratory of Resource Biology and Biotechnology in Western China, College of Life Sciences, Ministry of Education, Northwest University, Xi'an, China; ^2^Physiology Unit, Otto Loewi Center of Research for Vascular Biology, Immunity and Inflammation, Medical University of Graz, Graz, Austria

**Keywords:** disuse muscle atrophy, mechanical unloading, protein synthesis, protein degradation, molecular and cellular pathways, therapeutic countermeasures

## Abstract

Prolonged periods of skeletal muscle inactivity or mechanical unloading (bed rest, hindlimb unloading, immobilization, spaceflight and reduced step) can result in a significant loss of musculoskeletal mass, size and strength which ultimately lead to muscle atrophy. With advancement in understanding of the molecular and cellular mechanisms involved in disuse skeletal muscle atrophy, several different signaling pathways have been studied to understand their regulatory role in this process. However, substantial gaps exist in our understanding of the regulatory mechanisms involved, as well as their functional significance. This review aims to update the current state of knowledge and the underlying cellular mechanisms related to skeletal muscle loss during a variety of unloading conditions, both in humans and animals. Recent advancements in understanding of cellular and molecular mechanisms, including IGF1-Akt-mTOR, MuRF1/MAFbx, FOXO, and potential triggers of disuse atrophy, such as calcium overload and ROS overproduction, as well as their role in skeletal muscle protein adaptation to disuse is emphasized. We have also elaborated potential therapeutic countermeasures that have shown promising results in preventing and restoring disuse-induced muscle loss. Finally, identified are the key challenges in this field as well as some future prospectives.

## Introduction

Skeletal muscle is composed of muscular fibers and fascicles. It possesses a variety of functions in an organism's body and plays a vital role in the regulation of body metabolism. Skeletal muscles may differ significantly in mass, size, shape, and arrangement, depending upon their location and physical function in the organism. Elevated physical activity, like exercise, leads to increase the muscle mass (Bogdanis, 2012). On the other hand, decreased or limited use of skeletal muscle is one of the greatest contributing factors leading to muscle atrophy. Muscle atrophy may occur in a variety of conditions in unhealthy and healthy individuals, e.g., many common illnesses (Evans, 2010) including diabetes (Bonaldo and Sandri, 2013), cancers (Stephens et al., 2010), renal/heart failure, sepsis (Gordon et al., 2013), muscle genetic diseases (Sandri, 2010) and neurodegenerative disorders (Verdijk et al., 2012) cause significant loss of muscle mass. While in healthy individuals, muscle atrophy can also occur during conditions like spaceflight, bed rest, reduced step, hindlimb unloading (HLU) and immobilization. Furthermore, aging is also associated with muscle loss (Keller and Engelhardt, 2013; Hughes et al., 2017). Over the years, disuse skeletal muscle atrophy has been widely studied in humans as well as in animals.

Low skeletal muscle mass, decreased muscle fiber cross-sectional area (mCSA), muscle fiber transition from slow to fast and the change of functional properties have been observed in different muscle types under disuse conditions (Booth and Gollnick, 1983; Baldwin, 1996; Baldwin and Haddad, 2001; Fitts et al., 2001; Ohira et al., 2006). Several studies indicated that bed rest and immobilization cause disturbances in protein turnover (Goldspink, 1977; Janssen et al., 2000; Phillips et al., 2009; Rennie, 2009). It is commonly believed that a disproportion in the rate of protein synthesis and protein degradation is the main cause of muscle loss (Boonyarom and Inui, 2006; Bialek et al., 2011).

This review provides a summary of disuse muscle atrophy. In the first section, the functional and structural adaptations or alterations of skeletal muscle to disuse are discussed in different unloading models such as spaceflight, head down bed rest (HDBR), immobilization and reduced step in humans as well as HLU and immobilization in animals. The second section of the review provides a detailed discussion of anabolic and catabolic pathways and potential triggers involved in muscle protein synthesis and degradation under disuse-induced muscle atrophy. These include insulin-like growth factor-1-protein kinase B-mammalian target of rapamycin (IGF-1-Akt/PKB-mTOR), muscle ring finger 1/muscle atrophy F-box (MuRF1/MAFbx) and forkhead family of transcription factors (FOXO) pathways. The third section discusses the efficiency of potential countermeasures (antioxidants, resistance exercises and protein supplements) to counteract the loss of skeletal muscle.

## Mechanical unloading models

Spaceflight, bed rest, immobilization (cast/leg brace), step reduction and HLU are the key models extensively used to study the muscle loss in humans as well as in animals. These models provide deeper insights into the molecular and cellular mechanisms underlying disuse-induced muscle atrophy.

### Human models of disuse-induced muscle atrophy

The primary models of disuse in human research include microgravity induced muscle changes during spaceflight (Fitts et al., 2010; Goswami, 2017), bed rest immobilization (Spector et al., 2009), as well as other forms of immobilization (cast or leg brace) (Wall et al., 2013) and step reduction (Breen et al., 2013; McGlory et al., 2017). The majority of the published results of spaceflight experiments indicate that significant declines in skeletal muscle size, volume, CSA and strength occur after exposure to microgravity. It appear that longer sojourns in space lead to further atrophy and weakening of the muscles: 115–197 days in space were associated with significant decreases in muscle volume of gastrocnemius 17%, soleus 17%, and quadriceps 10% (LeBlanc et al., 2000). Another study also reported that prolonged spaceflight (approximately 180 days) significantly reduced force and fibers size in the gastrocnemius and soleus muscles. Specifically, the atrophy order (greatest-least) was: atrophy in soleus type I > soleus type II > gastrocnemius type I > gastrocnemius type II (Fitts et al., 2010). Furthermore, two long term spaceflight studies (140 and 175 days) reported a considerable decrease in characteristics of gastrocnemius muscle strength (Kozlovskaya et al., 1981). Similarly, 6 months Mir-mission in space found that isometric maximal voluntary contractions of the triceps surae muscle and peak tetanic force (Po) decreased by 42 and 25%, respectively (Koryak, 2001). In addition, short term spaceflight studies have also been reported to cause muscle weakness. For instance, volume changes in the knee extensor, knee flexor and plantar flexor muscle ranged from −15.4 to −5.5, −14.1 to −5.6 and −8.8 to −15.9, respectively, after 2 weeks in spaceflight (Akima et al., 2000). Edgerton and colleagues found that the size of all muscle fiber types of the vastus lateralis (VL) muscle decreased after 5–11 days of spaceflight (e.g., type I 16%, IIa 23%, and IIb 36%) and the percentage of type I myofibers decreased 6–8% (Edgerton et al., 1995). For instance, up to 8% decrease in CSA of the knee extensor and the gluteal muscles as well as 10% decrease of strength were observed after 17 days of spaceflight (Tesch et al., 2005).

In addition, differential rates of muscle atrophy have also been observed in response to disuse induced by HDBR in different muscle and fiber types. For example, prolonged bed rest with a 6° HDBR is one of the commonly used methods to mimic the effects of microgravity on muscle and bone turnover (Pavy-Le Traon et al., 2007; Spector et al., 2009). It was repeatedly demonstrated that HDBR leads to significant reduction in muscle strength and mass. Using magnetic resonance imaging (MRI), up to 17 and 40% loss in VL muscle volume and function were seen following 84-day HDBR (Trappe et al., 2004). Another 90-day study of HDBR reported up to 26% reduction in mCSA in young, healthy males (Rittweger et al., 2005). Indeed, similar results were obtained following 35 days of bed rest study (e.g., type I fiber CSA of VL showed greater loss than type II fiber) (Brocca et al., 2012). Similarly, a short duration HDBR study of 17–20 days also showed a 10–12% decrease in muscle size (Akima et al., 1997, 2001). On the other hand, Miokovic and associates observed that during prolonged bed rest, intramuscular differential atrophy did occur in most muscles, but some muscles of the lower limb remained unaffected (Miokovic et al., 2012).

In addition to bed rest, various other forms of immobilization have been used in ground-based studies of disuse muscle atrophy. Two week limb immobilization (casting) studies on young males reported reduction in quadriceps muscle volume, mCSA and strength by 9, 5–8, and 23%, respectively (Glover et al., 2008; Suetta et al., 2009). Even some shorter duration disuse studies have also reported significant reductions in muscle size (Wall et al., 2014). A five day study reported a 9% reduction in strength and 4% in quadriceps CSA (Dirks et al., 2014). Furthermore, immobilization appeared to impact the knee extensors to a greater degree than knee flexors (Veldhuizen et al., 1993; Deschenes et al., 2002). In addition to the above models of disuse, reduced step model has been demonstrated to influence muscle functions (Olsen et al., 2008; Knudsen et al., 2012; Breen et al., 2013). For example, reduced daily ambulatory activity has shown to lead to 2.8% loss in lean leg mass (Krogh-Madsen et al., 2010). Further data have shown that loss in muscle mass through lower limb disuse is more pronounced in older individuals (as they show increased vulnerability to loss of muscle size and strength) compared to younger individuals (Trappe, 2009; Degens and Korhonen, 2012; Tanner et al., 2015). It was observed in older participants (aged 68 ± 5 years) that 10-day bed rest leads to 7% reductions in muscle mass (lean tissue) (Kortebein et al., 2006). These findings were consistent with the results from a 7-day bed rest study involving six 60–73-year old participants, which reported 3.0 and 4.1% loss in total lean mass and lean leg mass, respectively (Drummond et al., 2012). Moreover, it has been observed that both contractile rate of force (torque) development and maximal isometric muscle strength are reduced significantly in the elderly as compared to younger men after 2 weeks of unilateral leg casting (Hvid et al., 2010). The discussed differences between young and old subjects are correlated with the effects of age-associated muscle atrophy (sarcopenia). Sarcopenia has been defined specifically as related to a subgroup of older persons with muscle-mass depletion, whose appendicular skeletal muscle mass (kg)/height^2^ (m^2^) is less than two standard deviations below the mean of a young reference group (Baumgartner et al., 1998). The muscle loss of sarcopenia has been attributed to the reduction in muscle fiber size (predominately in type II) and the muscle fiber number (Nilwik et al., 2013). Further detailed characteristics, mechanisms and functional significance of sarcopenia have been discussed in previous reviews (Evans, 2010; Narici and Maffulli, 2010).

From the foregoing discussion, it can be seen that the effects of several models of disuse (spaceflight, HDBR, immobilization and reduced step) on loss of muscle mass and strength have been widely investigated. Not all the studies have, however, reported consistent findings. This could be due to the fact that in most of these studies, either the study was restricted to investigation of only one muscle type or the immobilization was of short duration (≤14 days). Therefore, it is difficult to determine the details regarding differential rate of atrophy from these studies. Additionally, an important limiting factor in human studies is that most of the available data on atrophy and fiber CSA are based on a small biopsy sample taken from a single site.

### Animal models of disuse-induced muscle atrophy

Among all models of disuse-induced muscle atrophy, HLU and immobilization are, by far, the most widely employed animal models to study skeletal muscle atrophy in small mammals (Fitts et al., 1989; Morey-Holton and Globus, 2002; Caron et al., 2009). Generally, disuse muscle atrophy in rodents results in a rapid loss of muscle mass as well as in fiber CSA and function (within 14 days of unloading; Bodine, 2013). In the following section, we will attempt to evaluate the effectiveness of these two models with specific regards to the adaptations that are thought to occur in skeletal muscle.

The rodent HLU model is extensively used to simulate the physiological effects of microgravity (Morey-Holton and Globus, 2002; Lawler et al., 2003; Baehr et al., 2016). Most rodent studies focused on the slow soleus muscle, as it showed rapid atrophy (Wang et al., 2006; Sandonà et al., 2012). After 14 days of HLU 34-50% decrease of soleus muscle wet weight and 49% CSA were observed in rats (Ohira et al., 1992; Zhang et al., 2017). Similarly, another study indicated that electromyography of soleus was reduced at the start of unloading but then recovered fully within a week but muscle atrophy continued to increase (Ohira et al., 2015). Furthermore, fast type muscles (including gastrocnemius, plantaris and tibialis anterior but not extensor digitorum longus) also showed significant reduction in muscle mass following HLU (Tsika et al., 1987; Kyparos et al., 2005).

Another key model is limb immobilization, in which the desired part of the animal is covered with a plaster bandage or with a spiral wire and surgical skin staplers, which helps to maintain the joint in a particular position (Caron et al., 2009; Du et al., 2011). It is now well documented in rodents that soleus muscle wet weight and muscle fiber diameters for type I and II significantly decreased after 4 weeks of immobilization (Okita et al., 2001). It should be noted that muscle atrophy varies significantly under different conditions of immobilization. For example, it depends upon the position in which the joint is fixed/immobilized. Additionally, it was reported that muscles immobilized in short positions favor atrophy (Onda et al., 2016). Some studies also reported that the extensor muscles atrophy more than flexor muscles during ankle-joint immobilization (Roy et al., 1991; Adams et al., 2003; Ohira et al., 2006). The degree of skeletal muscles atrophy also showed differential responses in regards to the types of fiber (Jozsa et al., 1988). For example, 4 week hindlimb immobilization studies on male rats reported that type I fibers of the soleus muscle undergo greater reductions than type II fibers (Booth and Kelso, 1973; Thomason and Booth, 1990) and similar results have also been obtained during ankle-joint immobilization (Thomason and Booth, 1990; Ohira et al., 2006).

Taken together, varying results among different studies in rodent models of HLU and limb immobilization are related to the muscle type, muscle fiber type and conditions of immobilization. It has been shown that the amount of muscle loss is greater in the extensor muscles of the ankle (soleus and gastrocnemius) as compared to the flexor muscles (tibialis anterior and extensor digitorum longus) (Ohira et al., 2002; Adams et al., 2003; Zhong et al., 2005). Additionally, muscle atrophy appears to be different across muscle types. For example, slow-twitch (type I) fibers are more vulnerable and therefore, show a greater loss in the amount of protein than fast twitch (type II) fibers (Tsika et al., 1987; Thomason and Booth, 1990; Zhang et al., 2015), and the muscles immobilized in short positions showed more sensitivity to disuse (Desaphy et al., 2010). These observations suggest that the rate and extent of muscle loss appear to depend on the degree of unloading, the extent of physical inactivity and the muscle type.

## Mechanisms of muscle atrophy

Alterations and underlying mechanisms of muscle protein synthesis and degradation have been investigated extensively in different disuse models (Bodine, 2013; Bonaldo and Sandri, 2013; Rudrappa et al., 2016). In addition, some new insights and findings also have been reported recently (Mirzoev et al., 2016; Baehr et al., 2017). In this section, we provide a comprehensive summary of the molecular basis of disuse atrophy including potential triggers to signaling pathways and their ultimate effects on the myofibrillar apparatus.

### Protein synthesis and disuse atrophy

It has been well recognized that decreased protein synthesis in muscle seems to be the major contributor in disuse muscle atrophy (Bodine, 2013). The decline of the basal protein synthesis rate in the early period of unloading has been investigated and confirmed in human and animal models (Booth and Seider, 1979; de Boer et al., 2007; Mirzoev et al., 2016; Baehr et al., 2017). Thus, the underlying mechanisms of decreased protein synthesis in disused skeletal muscle have been a main focus research area the field for the past few decades. Although many problems have not been solved yet, recent research has confirmed that decreased activation of the Akt-mTOR pathway is involved in the mechanisms of the attenuated protein synthesis under disuse conditions. In the following section, we give the details about this pathway both in normal and unloading conditions.

**Figure 1:**
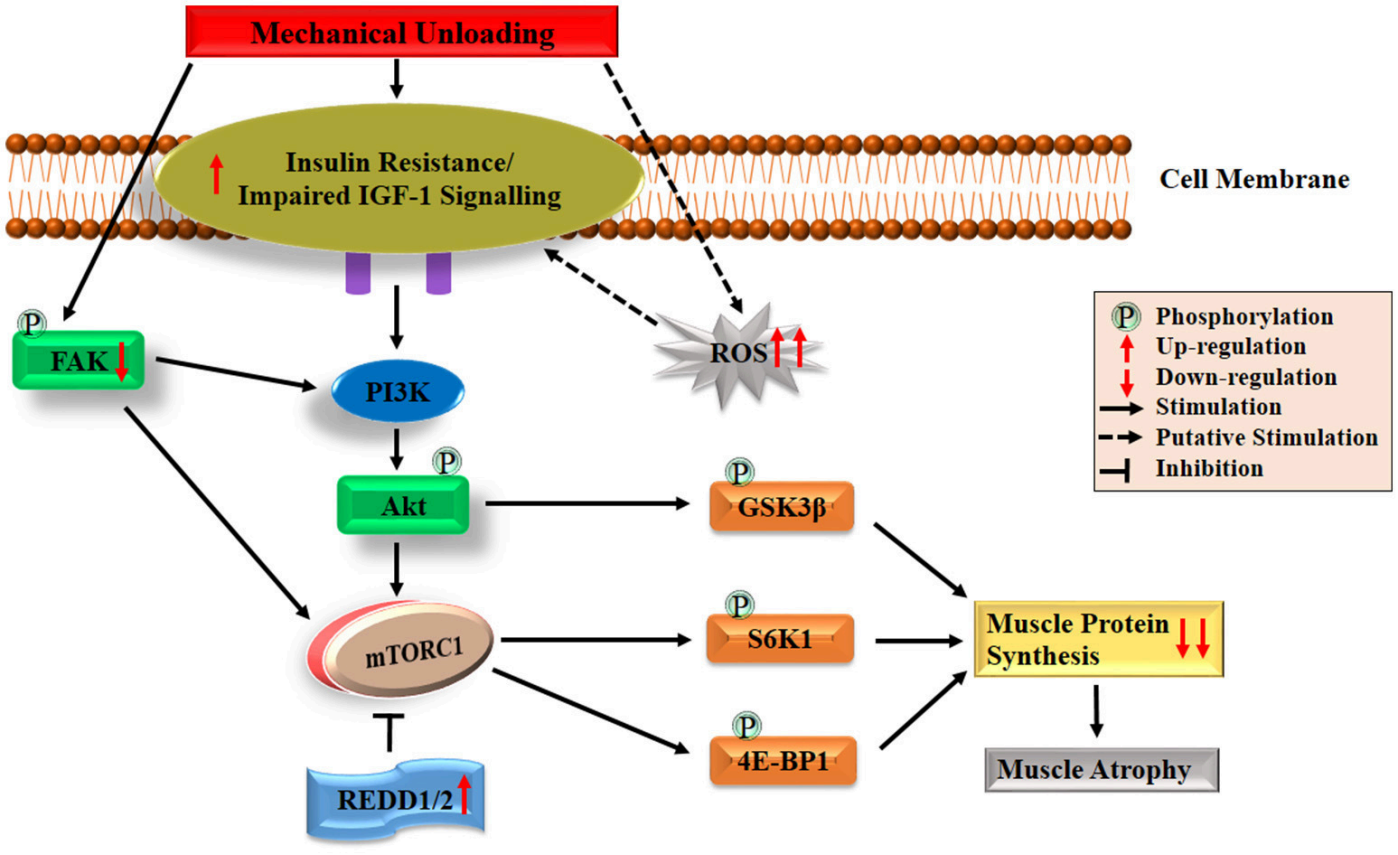
Diagrammatic representation of the protein synthesis signaling mechanisms responsible of skeletal muscle atrophy following mechanical unloading. IGF-1, insulin-like growth factor-1; PI3K, phosphatidylinositol 3 kinase; Akt/PKB, protein kinase B; FAK, focal adhesion kinase; mTORC1, mechanistic target of rapamycin in complex 1; REDD, regulated in DNA damage and development; GSK-3β, glycogen synthase kinase 3β; S6K1, 70 kDa ribosomal protein S6 kinase 1; 4E-BP1, eIF4E binding protein 1; ROS, reactive oxygen species. The black arrow and inhibition symbol show the association of molecules under loading condition, red arrow shows the up/down-regulation of molecules under unloading condition. For more details see text.

Under normal physiological condition, the IGF-1-Akt-mTOR pathway acts as a key regulator in the translation initiation step of protein synthesis in skeletal muscle (Erbay et al., 2003; Han et al., 2008; Schiaffino and Mammucari, 2011). Firstly, the IGF-1-Akt-mTOR pathway is initiated by binding of the IGF-1 to its specific IGF-1 receptor (IGF-1R), which triggers a signaling cascade mainly stimulating the intrinsic tyrosine kinase activity through insulin receptor substrate-1 (IRS-1). Subsequent activation of phosphatidylinositol 3 kinase (PI3K) may be achieved by binding p85 regulatory subunit with phosphorylated IRS-1 (Jheng et al., 2012). The membrane phospholipid-phosphatidylinositol-4,5-bisphosphate (PIP2), is phosphorylated into phosphatidylinositol-3,4,5-triphosphate (PIP3) by PI3K. Then PIP3 recruits phosphoinositide-dependent protein kinase-1 (PDK1) to phosphorylate and activate Akt. Phosphorylated Akt further activates mTORC1 (Inoki et al., 2002; Goodman et al., 2010; Miyazaki et al., 2011). Consequently, activated mTORC1 phosphorylates both 4E-BP1 and S6K1 which finally leads to protein synthesis (Gordon et al., 2013). Besides the indirect pathway by mTORC1, activated Akt can also phosphorylate glycogen synthase kinase 3β (GSK-3β), which directly leads to the increase of global protein synthesis through an increased activity of eukaryotic initiation factor 2B (eIF2B) (Welsh et al., 1998). The simplified version of the regulating mechanism underlying IGF-1-Akt-mTOR pathway on muscle protein synthesis is shown in Figure 1.

Under unloading conditions, insulin resistance plays an important role in driving depression of protein synthesis. This has been widely observed in humans subjected to bed rest confinement (Shangraw et al., 1988; Stuart et al., 1988; Hamburg et al., 2007), immobilization (Richter et al., 1989) and HLU in animals (Allen et al., 1997). Research on disuse models of rodents (e.g., HLU, immobilization and denervation) showed that insulin resistance induced attenuation of Akt-mTORC1 pathway may provide a mechanism for decreased protein synthesis (Gordon et al., 2013). Reduced activation of this pathway which characterized as decreased phosphorylation of Akt, S6K1, and 4E-BP1 has been shown in the soleus and medial gastrocnemius muscles (Bodine et al., 2001b; Hornberger et al., 2001; Sugiura et al., 2005; Haddad et al., 2006; Kelleher et al., 2013). Liu and colleagues also found that the binding of 4E-BP1 and eIF4E altered in rat gastrocnemius muscle during the early period of HLU (Liu et al., 2012). In addition, decreased phosphorylation of GSK3β has been observed in HLU rats (Stevenson et al., 2003; Mirzoev et al., 2016). Recent studies have also reported that an important mTOR signaling repressors such as mRNA expressions of regulated in DNA damage and development 1 and 2 (REDD1/2), significantly elevated following unloading in rats (Kelleher et al., 2013, 2015). Moreover, it has been reported that both Akt-null and mTOR knockout mice exhibited significant skeletal muscle atrophy as well as growth deficiency, which also proved the essential role of Akt-mTOR pathway on muscle maintenance (Peng et al., 2003; Risson et al., 2009). All the above discussion demonstrates the essential role of Akt-mTOR pathway in animals, but its role in controlling muscle protein synthesis in humans unloading models remains unclear. For example, human immobilization studies reported decrease in protein synthesis rate, but no changes were observed in Akt-mTORC1 signaling pathway (de Boer et al., 2007; Glover et al., 2008; Marimuthu et al., 2011). This suggests that decreased protein synthesis rate could also be regulated through other signaling pathways in human. Among other possible pathways, the most noteworthy one is focal adhesion kinase (FAK), a mechanosensitive non-receptor protein tyrosine kinase, located in the costamere region of skeletal muscle fibers and is sensitive to changes in mechanical loading (Bloch and Gonzalez-Serratos, 2003; Anastasi et al., 2008). Some crosstalk has been reported between FAK and PI3K-Akt-mTOR pathway. On the one hand, phosphorylation of tyrosine 397 of FAK results in the binding of FAK to the SH2 domain of the 85 kDa subunit of PI3K, which can lead to the increase in PI3K activity and subsequently activate Akt-mTOR pathway (Chen et al., 1996). On the other hand, activated FAK may upregulate mTOR through inhibiting TSC2 (a negative regulator of mTOR) by phosphorylation (Graham et al., 2015). Under physical inactivity conditions, reduced phosphorylation of FAK was discovered in humans and animals, which suggests that attenuated activation of FAK-Akt-mTOR is another key contributor to the decreased protein synthesis during atrophy condition (de Boer et al., 2007; Glover et al., 2008; Graham et al., 2015). Collectively, both the declined activation of IGF1–Akt–mTOR pathway induced by impaired IGF-1 signaling/insulin resistance and the decreased activation of FAK-Akt-mTOR pathway caused by reduced FAK phosphorylation play essential roles in regulating skeletal muscle protein synthesis during atrophy conditions. The possible mechanisms are also summarized briefly in Figure 1.

In addition to the regulation of translation initiation, it has also been reported that eukaryotic elongation factor 2 (eEF2) plays an important role in the regulation of protein synthesis at the level of elongation of mRNA translation process (Redpath et al., 1996). Recently it was observed that the level of eEF2 phosphorylation (inactive form) in soleus muscle elevated significantly after 14 days of HLU in rats (Lomonosova et al., 2017). Besides, protein synthesis also depends on translational capacity, the main component of which is the number of ribosomes (McCarthy and Esser, 2010; Chaillou et al., 2014). Decreases in the content of both total RNA and 28S rRNA (one of the key markers of ribosome content) were observed after 1, 3, 6, and 7 days of HLU in rat soleus (Bajotto et al., 2011; Mirzoev et al., 2016). Although great progress has been made as described above, there is still a lot of work that needs to be done. For example, studies with frequent biopsy sampling are required to comprehensively understand the role of mTORC1 signaling in regulating the depression in postprandial and post-absorptive muscle protein synthesis, especially in human models. In addition, more research is needed to clarify the relative downstream genes for regulation of ribosome assembly in the Akt-mTOR pathway.

### Protein degradation and disuse atrophy

**Figure 2:**
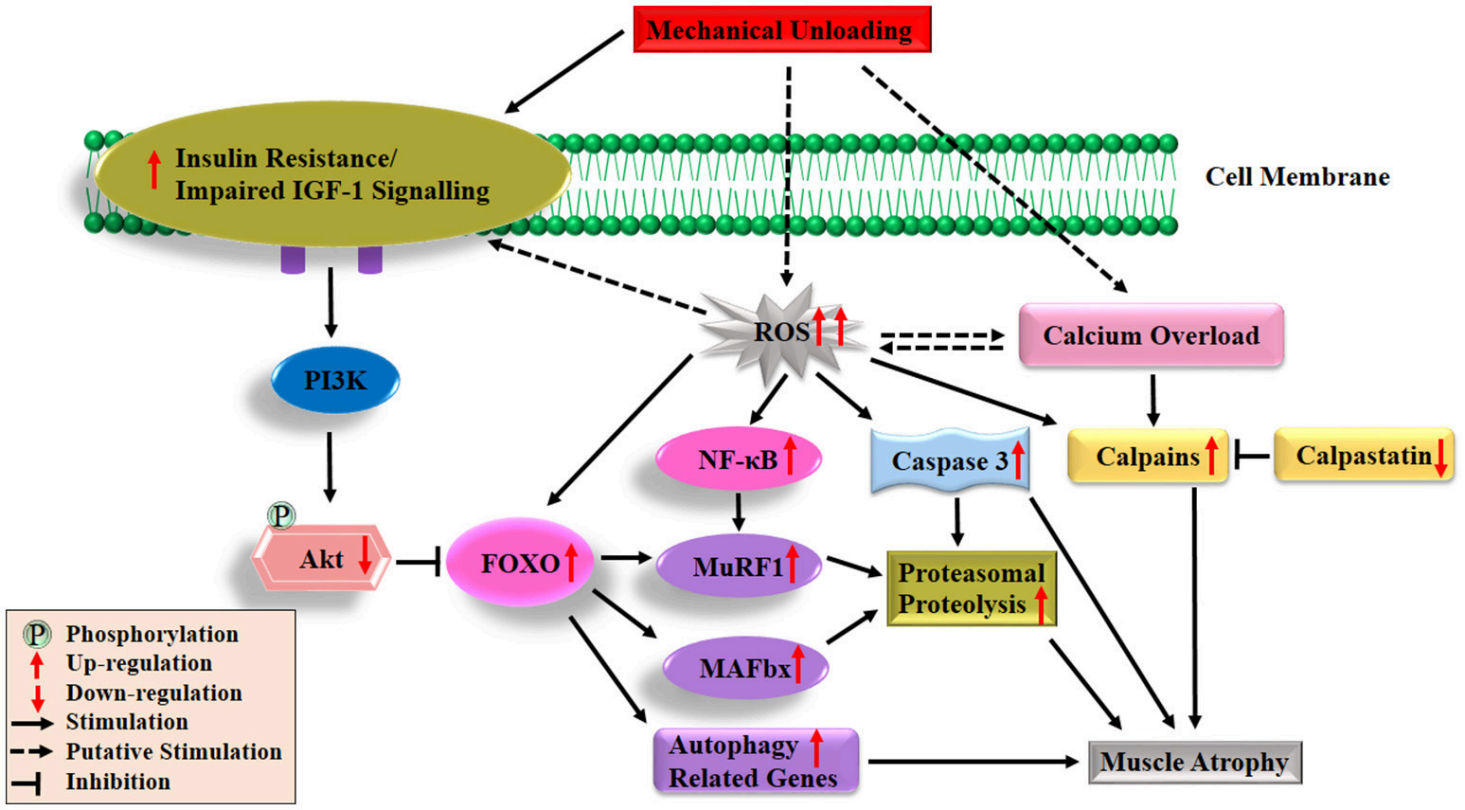
Diagrammatic representation of the protein degradation signaling mechanisms responsible of skeletal muscle atrophy following mechanical unloading. IGF-1, insulin-like growth factor-1; PI3K, phosphatidylinositol 3 kinase; Akt/PKB, protein kinase B; FOXO, forkhead family of transcription factors; NF-κB, nuclear factor kappa-B; MuRF1, muscle ring finger 1; MAFbx, muscle atrophy F-box 1; ROS, reactive oxygen species. The black arrow and inhibition symbol show the association of molecules under loading condition, red arrow shows the up/down-regulation of molecules under unloading condition. For more details see text.

In contrast to the recognized deficits in muscle protein synthesis during disuse conditions, the role of protein breakdown in disuse-induced muscle atrophy is less clear. This is partly due to the lack of direct measurements of muscle protein degradation in studies. Instead, indirect studies of protein degradation pathways have to be employed to measure molecular markers of muscle proteolysis. The major protein degradation pathways in skeletal muscle include the Ca^2+^-dependent proteases, lysosomal system, caspases and ubiquitin proteasome pathways (Scicchitano et al., 2015). It was proposed that Ca^2+^-dependent proteases (calpains) act as a promoter of muscle protein degradation, and might be responsible for the discharge of myofilaments from the surface of myofibrils (Dayton et al., 1976). Subsequently, the myofilaments were ubiquitinated and degraded to amino acids by proteasome intracellular peptidase cathepsins (Figure 2; Huang and Zhu, 2016). Emerging evidence suggests that calpains (Huang and Zhu, 2016), caspase-3 (Talbert et al., 2013), autophagy-lysosomal system (Sandri, 2010) and ubiquitin proteasome pathway (Bodine and Baehr, 2014), all are involved in disuse-induced muscle atrophy. However, the ubiquitin proteasome system is often considered as the most important proteolytic system during disuse conditions that promotes muscle wasting (Scicchitano et al., 2015; Baehr et al., 2017). The breakdown of protein via the ubiquitin proteasome system requires three distinct enzymatic components of the ubiquitin proteasome pathway: E1 (ubiquitin-activating enzyme), E2 (ubiquitin-conjugating enzyme) and E3 (ubiquitin ligase, key enzyme which regulates proteolysis as it recognizes multiple target protein substrates). Two muscle specific classes of E3s (MuRF1 and MAFbx/atrogin-1) have been studied widely and play an essential role during skeletal muscle atrophy (Mitch and Goldberg, 1996; Foletta et al., 2011; Bodine and Baehr, 2014). In various animal models of disuse muscle atrophy (HLU, immobilization, spaceflight and denervation), mRNA levels of both genes (MuRF1/MAFbx) were rapidly increasing and thought to play a crucial role in the initiation of the atrophy process (Bodine et al., 2001a; Lecker et al., 2004; Murton et al., 2008; Allen et al., 2009; Baehr et al., 2017; Gambara et al., 2017). In addition, it has been reported that the degree and the time course of the upregulation of these two genes is not uniform among different muscles. For example, a more significant increase in the expression of MuRF1/MAFbx genes occurred in ankle plantar flexors (soleus and medial gastrocnemius) than dorsi flexors (tibialis anterior), and there was a longer duration of expression in ankle plantar flexors than dorsi flexors in response to unloading (Bodine et al., 2001a; Lecker et al., 2004). Moreover, studies using MAFbx and MuRF1-deficient mouse models further supported a potential contributing role of these two genes in the development of muscle atrophy. For instance, mice deficient in either MAFbx or MuRF1 were found to be resistant to atrophy (Bodine et al., 2001a). Another study also indicated remarkable protection of the soleus muscle in the MuRF1-KO mice during 10 days of HLU (Labeit et al., 2010). It should be noted that various reports have been shown about MuRF1 and MAFbx during disuse-induced atrophy in human models (Murton et al., 2008). For example, both the MuRF1 and MAFbx mRNAs increased significantly after 2 days (Abadi et al., 2009) and 5 days of immobilization (Dirks et al., 2014) or after 3 days of unilateral lower limb suspension (ULLS) (Gustafsson et al., 2010). However, in a 20-day bed rest study, elevated MAFbx but not MuRF1 mRNA were observed in VL (Ogawa et al., 2006), same results were observed during 14 days of leg immobilization (Jones et al., 2004). On the other hand, de Boer and colleagues reported that increased MuRF1, mRNA expression, but not MAFbx were observed during 0–10 days of immobilization (de Boer et al., 2007). In addition, it has been reported that MuRF1 protein expression increased in soleus, but not in VL after 60 days of bed rest (Salanova et al., 2008).

Under disuse conditions, it has been reported that expression of E3 ubiquitin ligases MuRF1 and MAFbx are regulated by various upstream factors or signaling pathways (Bodine and Baehr, 2014). FOXO transcription factors (FOXO1 and FOXO3) have been stated as the major transcription factors regulating both the MuRF1 and MAFbx expressions (Sandri et al., 2004; Stitt et al., 2004). It is noteworthy that the functional aspects of FOXO are determined by their cellular location and predominantly regulated by the IGF-1-PI3K-Akt pathways (Brunet et al., 1999; Zhao et al., 2007). Under normal physiological conditions, Akt phosphorylates FOXO on specific threonine and serine residues, which results in the retention of FOXO in the cytosol instead of translocating to the nucleus (Brunet et al., 1999). Under muscle disuse conditions, dephosphorylated FOXO translocates to the nucleus and up-regulates several different types of atrogenes (E3 ligases) or autophagy related genes as shown in Figure 2 (Stitt et al., 2004). Several studies reported that mRNA and protein levels of FOXO1 and/or FOXO3 expression in the slow-twitch soleus muscle, mixed fiber type gastrocnemius muscle and fast twitch plantaris muscle are upregulated following different muscle atrophy condition (Giresi et al., 2005; Sacheck et al., 2007; Allen et al., 2009; Levine et al., 2011; Okamoto and Machida, 2017). Sandri and colleagues found that constitutively active FOXO3 acts on the MAFbx promoter to cause MAFbx transcription and dramatic atrophy of myotubes and muscle fiber. It further suggested that stimulation of the two main proteolytic pathways (MuRF1/MAFbx) via overexpression of FOXO1 and FOXO3 leads to a reduction in muscle mass and strength during disuse inactivity (Sandri et al., 2004). Direct or indirect inhibition of FOXO transcriptional activity or suppressed expression of co-factors (MuRF1/MAFbx) and interaction with other transcription factors lead to the attenuation of disuse-induced muscle atrophy (Senf et al., 2008; Reed et al., 2012; Brocca et al., 2017). But these two transcription factors mRNA are not always linked and that changes in the expression levels of both genes (MuRF1/MAFbx) are dependent on the muscle and the time after unloading (Atherton et al., 2016). Interestingly, it was observed that constitutively active FOXO3 controls the stimulation of autophagic/lysosomal proteolysis pathway, thus leading to muscle wasting in fasting and denervation models (Mammucari et al., 2007). Put-together, these findings strongly support the notion that most of these transcription factors and signaling pathways play important roles in the progression of muscle disuse atrophy. Remarkably, increased mRNA levels of both genes (MuRF1/MAFbx) were observed in animals following disuse conditions as shown in Figure 2. While, data were inconsistent in human models, a previous review showed that activation of these two genes mainly occurs in muscle wasting caused by inflammation (e.g., cancer, chronic obstructive pulmonary disease, severe head trauma, amyotrophic lateral sclerosis, critical illness, AIDS) with the data in non-inflammatory muscle atrophy is inconsistent (Narici and Maffulli, 2010). Only a few studies have measured the changes in ubiquitinated protein content and proteasomal proteolysis following disuse atrophy (Helliwell et al., 1998; Ogawa et al., 2006; Slimani et al., 2012; Baehr et al., 2016). There is a need for additional studies on human biopsies to explore the activity of ubiquitin-proteosome pathway components.

### Major triggers of disuse atrophy

Besides the above-mentioned pathways regarding muscle protein synthesis and degradation following disuse atrophy, there are two important factors contributing to protein turnover. The following discussion gives a review regarding the possible triggers reported during muscle disuse.

#### ROS and disuse atrophy

Oxidative stress, characterized by the increased reactive oxygen species (ROS) production and impairment of antioxidant defense systems, has frequently been observed in disuse and other pathological conditions. It is now widely considered as a major trigger of the imbalance between protein synthesis and degradation leading to muscle atrophy (Powers et al., 2005, 2007; Moylan and Reid, 2007; Powers, 2014; Zuo and Pannell, 2015). Production of ROS results in the inhibition of insulin action and acts as a putative mediator in the development of insulin resistance (Bashan et al., 2009; Di Meo et al., 2017). Furthermore, growing evidence indicates that oxidative stress can promote muscle protein breakdown through the following aspects. Firstly, oxidative stress, promotes expression of proteins involved in proteolytic pathways, such as autophagy, calpain and the ubiquitin–proteasome system of proteolysis. Secondly, oxidative stress results in the activation of two important proteases, calpain and caspase-3. Thirdly, increased ROS production in muscle fibers can also promote proteolysis by oxidative modification of myofibrillar proteins, which enhances their susceptibility to proteolytic processing. The details about the three aspects have been discussed in a previous published review (Powers, 2014). ROS also might be the upstream activators of nuclear factor kappa-B (NF-κB) and FOXO pathways in skeletal muscle atrophy (Dodd et al., 2010). The roles played by ROS in protein synthesis and degradation are depicted in Figures 1, 2, respectively.

#### Calcium overload and disuse atrophy

Calcium (Ca^2+^) is necessary to carry out many important body functions such as cell metabolism, cardiac and skeletal muscle contraction, tissue differentiation and neurotransmission (Zhou et al., 2013). Ca^2+^ and endogenous inhibitor calpastatin are the two major regulators on calpains activation during disuse conditions (Figure 2; Huang and Zhu, 2016). Previous studies reported highly elevated cytosolic free Ca^2+^ concentration in soleus and gastrocnemius muscles during disuse conditions (Ingalls et al., 2001; Xu et al., 2012; Hu et al., 2017). Two ubiquitous calpains, calpain1 and calpain2 (also called u- and m-) are activated by elevated intracellular Ca^2+^ in HLU rats (Matsumoto et al., 2014; Zhang et al., 2017). In addition, caspase-3-dependent apoptosis, another major signaling pathway involved in disuse muscle atrophy (Talbert et al., 2013), is also activated by intracellular Ca^2+^ overload through two distinct pathways. On the one hand, the intracellular Ca^2+^ overload leads to the activation of caspase-12 which then activates caspase-3 (Primeau et al., 2002). On the other hand, increasing Ca^2+^ levels induces activation of pro-apoptotic protein Bax, which translocated and inserted into the outer membrane of mitochondria via forming Bax/Bax-homo-oligomerization. Bcl-2, another Bcl-2 family protein, could inhibit the formation of Bax/Bax-homo-oligomerization. The decline of the ratio of Bax/Bcl-2 leads to the release of pro-apoptotic factors from the mitochondria, which subsequently activates caspase-9 and caspase-3 (Zha et al., 1996; Antonsson et al., 1997; Chen et al., 2002; Garrido et al., 2006). In one of our previous studies, the increase of Bax/Bcl-2 and cytochrome C release were observed in gastrocnemius in rats following 14-day HLU (Hu et al., 2017). Collectively, both the overproduction of ROS and Ca^2+^ overload play an essential role in regulation of protein synthesis and degradation following disuse conditions as shown in Figure 2. However, the relationship between ROS and Ca^2+^ remains unclear and more research is needed to clarify this relationship.

In addition to the anabolic and catabolic pathways mentioned above, recently emerging evidence indicates some other key factors, such as P53, activating transcription factor 4 (ATF4), growth arrest and DNA damage-inducible 45a protein (Gadd45a) and P21, are significantly elevated under disuse conditions (Ebert et al., 2012; Fox et al., 2014; Bullard et al., 2016). Therefore, we recommend that the reader consult the most recently published reviews on the topic (Brooks and Myburgh, 2014; Adams et al., 2017). Taken together, during disuse conditions, both the protein synthesis and degradation play an essential role during muscle atrophy, and in particular, suppressed protein synthesis has been confirmed. However, more research has to be carried out to clarify the details mechanisms of protein degradation and insulin resistance in driving disuse-induced muscle atrophy.

## Therapeutic countermeasures

Various therapeutic interventions, pharmaceutical options and rehabilitation programs have been used to prevent and limit the loss of skeletal muscle. These therapeutic countermeasures can be grouped into three categories: antioxidant and anti-inflammatory compounds, nutritional supplements and physical training and exercise.

### Antioxidant and anti-inflammatory compounds

It has been demonstrated that muscle damage, oxidative stress and inflammation have a negative impact on protein turnover of skeletal muscle, predominantly via decreases in protein synthesis (Peterson et al., 2011; Powers et al., 2012). Oxidative stress is thought to be one of the major factors leading to many health-related disorders including skeletal muscle dysfunction (Powers et al., 2012). In addition, increased mitochondria ROS production, as well as endoplasmic reticulum stress and decrease of antioxidant capacity, are three major factors that play key roles in triggering sarcopenia with aging (Drew et al., 2003; Short et al., 2005; Narici and Maffulli, 2010). Thus, antioxidants have been shown to prevent oxidative stress associated damage and have been proven to be effective countermeasures against muscle atrophy to maintain skeletal muscle mass and strength, especially in elders (Servais et al., 2007; Cornetti, 2009; Rieu et al., 2009; Stojiljkovic et al., 2016).

In recent years, polyphenols, a well-recognized antioxidants, have been studied extensively with regard to their roles in the prevention of neurodegenerative diseases/skeletal muscle atrophy. Resveratrol, is one of the naturally occurring polyphenols, well known for its great health benefits, and frequently found in berries, grapes, red wine and some other fruits and vegetables (Brito et al., 2008; Das et al., 2008). It has been suggested that resveratrol plays an important role in the transcription of two important antioxidant enzymes, i.e., Mn superoxide dismutase (SOD) and catalase (Dani et al., 2008; Kode et al., 2008; Robb et al., 2008; Ryan et al., 2010). Six month old adult rats were treated with resveratrol (12.5 mg/kg/day) for 5 weeks (including 2 weeks of muscle immobilization) with results indicating that it reduced the functional decrements and the oxidative stress level (Jackson et al., 2010). In another study, rats were supplemented with resveratrol at a dose (400 mg/kg/day) before unloading and 2 weeks of muscle immobilization. During treatment with resveratrol, muscle disuse atrophy was significantly reduced (Momken et al., 2011). These two studies strongly suggest that metabolic and muscle deconditioning in response to mechanical unloading can be prevented by the use of high dosage of the antioxidant, resveratrol. Similarly, in another study, mice were treated with tea catechins (comprising of up to 81% polyphenols) at a dosage of 46–50 mg/kg. The antioxidant diet was consumed before and during immobilization of 14 and 10 days, respectively. This study showed that the antioxidant (tea catechins) did not suppress muscle atrophy completely, but it helped in the maintenance of tetanic force observed in the soleus muscle in response to immobilization. This study also suggested that tea catechins have positive effects on skeletal muscle function rather than skeletal mass, and they help to improve muscle strength (Ota et al., 2011).

In addition to the above findings, several studies have reported the use of several other antioxidants [e.g., vitamin E or SS-31 (D-Arg-2′6′dimethylTyr-Lys-Phe-NH2) for the prevention of disuse muscle atrophy, (Servais et al., 2007; Powers, 2014)]. For instance, administration of vitamin E was shown to significantly reduce soleus muscle atrophy during 14-day HLU. The results of this research also demonstrated that the protective role of vitamin E does not depend on its antioxidant activity, but it might be due to alteration in muscle protein degradation (Servais et al., 2007). While, Koesterer and colleagues reported that vitamin E supplementation has no effect on HLU induced soleus and gastrocnemius muscle atrophy (Koesterer et al., 2002). In addition, it has been reported that SS-31, one of the essential mitochondrial-targeted antioxidant, could protect against HLU induced soleus and plantaris muscles atrophy both in rats and mice (Min et al., 2011; Talbert et al., 2013). Additionally, some authors reported that another two widely used antioxidants, either curcumin or N-acetylcysteine treatment could protect the diaphragm against ventilator-induced muscle wasting (Agten et al., 2011; Smuder et al., 2012), but did not prevent against inactivity-induced limb muscle atrophy (Farid et al., 2005).

In addition to the widely used antioxidant to prevent disuse atrophy, some anti-inflammatory compounds and pharmaceutical options were also adopted in this field. Some studies recommended the use of dietary fish oil to prevent immobilization-induced atrophy. Fish oils are well known for their anti-inflammatory properties as they contain different fatty acids (long chain n-3 fatty acid; Fetterman and Zdanowicz, 2009). N-3 fatty acids facilitate insulin-sensitive protein anabolism through the Akt-mTOR-S6K1 pathway, which prevents anabolic resistance and leads to decreased muscle atrophy induced by disuse (Gingras et al., 2007). Furthermore, another study reported that ingestion of 5% fish oil reduces disuse muscle atrophy via Akt pathway through E3 ubiquitin ligases and S6K1 pathway (You et al., 2010). Chromium (Cr) is also believed to preserve muscle mass by inhibiting the elevation of ubiquitin proteasome system pathway and restoring the impaired Akt signal through elevating the Akt phosphorylation (Dong et al., 2009).

Studies carried out in our laboratory show that tetramethylpyrazine is a dietary supplement that could effectively alleviate muscle atrophy in HLU rats (Gao et al., 2005; Zhang et al., 2007; Li et al., 2012). It was demonstrated that attenuating disuse-induced Ca^2+^ overload and activation of calpains system might be involved in the underlying mechanism of tetramethylpyrazine to counteract disuse-induced muscle atrophy (Wu et al., 2012; Hu et al., 2017; Zhang et al., 2017). Furthermore, various Chinese herbal medicines have also been used to reduce and prevent muscle loss caused by different unloading models. For example, *Sijunzi Decoction* (Hu et al., 2009), *Angelica Sinensis* (Qin et al., 2009; Du and Gao, 2014), *Ligusticum* (Qin et al., 2009), and *Radix Astragali* (Gao et al., 2005, 2008; Zhang et al., 2007) have been tested under different muscle atrophy conditions by our laboratory.

The majority of these traditional Chinese medicines have positive effects on blood circulation and/or are blood tonics have been reported to attenuate disuse-induced muscle atrophy (Gao et al., 2005; Zhang et al., 2007, 2017; Wu et al., 2012; Hu et al., 2017). Due to numerous effective components in these herbs, the major effective ingredients and the mechanisms involved against muscle atrophy remain unclear. Thus, future research should be carried out to identify specific and effective components of these herbs, thus leading to wider applications of these herbs to counter muscle atrophy.

### Exercise and physical training

Exercise is one of the best countermeasure against disuse atrophy. During physical inactivity conditions such as HDBR and HLU, exercise has been shown to be the most efficient countermeasure to address the deficits in muscle structure and function, as well as for maintenance of balance between muscle protein synthesis and protein breakdown (Herbert et al., 1988; Widrick et al., 1996; Shinohara et al., 2003). Loss in muscle mass during physical inactivity is more challenging in aged people compared to younger persons and, therefore, to maintain muscle protein synthesis rate, older individuals required more resistance exercise than younger individuals (Kumar et al., 2012). For instance, after 2 weeks of ULLS following 4 weeks of resistive exercise employed in younger and older individuals suggested that recovery of strength and muscle size was much more reduced in older adults (Suetta et al., 2009; Hvid et al., 2010). These findings were further supported by a study of older women in which resistance training of 12 weeks was performed (at a frequency of 3 times per week). These investigators observed an increase in quadriceps muscle volume by up to 6% in young women and only 3% increase in older women (Greig et al., 2011). Hvid and co-workers observed marked decrements in knee extensor muscle function in young and old individuals after 4-day lower limb disuse. Following 7-day recovery, knee extensor (isometric or isokinetic) strength was recovered in young individuals, while an impaired ability to fully recover was observed in older individuals (Hvid et al., 2014).

Moreover, it has been documented that different types of resistance exercises play a key role in the maintenance of muscle mass in disuse models (e.g., bed rest, HLU) by improving muscle protein synthesis via activation of the PI3K-Akt-mTOR pathway (Ferrando et al., 1997; Fluckey et al., 2004; Hornberger et al., 2004; Philp et al., 2011). It has been proposed that short period of resistance exercise can activate IGF-1 gene expression in healthy individuals (Chesley et al., 1992). For instance, full restoration of quadriceps muscle mass following 2 weeks of single leg immobilization in humans can be achieved via high-resistance training (Oates et al., 2010). Additionally, combination of endurance and resistance exercise is an effective modality to counter the muscle loss associated with disuse or inactivity in HLU mice (Adams et al., 2007). In fact, resistance exercise partly rescued the loss in cytoskeletal and dystrophin-associated glycoprotein in VL and soleus muscles at protein level for the duration of extended bed rest (84 days) in human beings (Chopard et al., 2005). It was also demonstrated that resistance exercises associated with a ~50% decrease in the stimulation of the ubiquitin-proteasome system (MuRF1/MAFbx) and alleviate muscle atrophy caused by 14 days of HLU (Dupont-Versteegden et al., 2006).

Moreover, a number of other published literatures opine that the increased mechanical load can activate cellular signaling that initiates the protein synthesis independent of a traditionally described functioning IGF-1 receptor (Bickel et al., 2005; Hornberger et al., 2006; Spangenburg et al., 2008; Hamilton et al., 2010; West et al., 2010; Witkowski et al., 2010; Gabriel et al., 2016). Noticeably, these experiments pave the way for future investigations regarding Akt-mediated signaling in response to mechanical loading and other growth stimuli, as well as provide new insights for the prevention of disuse-induced muscle atrophy.

In general, applying exercise and physical training is one of the most widely used, effective and with minimal side effects in all countermeasures, but exercise and physical training cannot always be applied to injured patients with fractures and is often problematic for bed rest patients. In addition, the acceptance of this countermeasure is also difficult for those who do not want to exercise. Thus, exercises that possess minimum load or minimum exercise time against disuse muscle atrophy needs further investigation.

### Nutrition and protein supplementation

Exercise alone cannot avert the disuse-induced muscle loss under different unloading conditions, including bed rest confinement during hospitalizations and sedentary lifestyle. Hence, several other approaches, such as nutritional supplementation (essential amino acid and protein), should be used in conjunction with exercise to rescue or counteract better against catabolic processes during chronic disuse. Specifically, muscle mass and strength can be more effectively enhanced by combining the nutritional regulation (dietary carbohydrates and amino acid) with an adaptive exercise regimen than by application of either treatment approach alone (Dreyer et al., 2008; Pasiakos et al., 2011). The use of protein supplement along with heavy resistance training can significantly improve muscle strength and mass in very old individuals (Bechshøft et al., 2017). Generally speaking, it appears that during immobilization and bed rest confinement, protein synthesis can significantly be improved by using amino acid supplementation, but it can only partially avert muscle atrophy (Glover et al., 2008). Amino acids are very well known for their important role in the regulation of protein synthesis and metabolism by accelerating the initiation step of peptide chain formation in skeletal muscle (Biolo et al., 1997; Bohé et al., 2001; Rennie et al., 2004; Stipanuk, 2007). It has consistently been shown that essential amino acid supplementation strengthens the response of muscle protein synthesis and partially rescues skeletal muscle loss experienced during bed rest (Stuart et al., 1990; Paddon-Jones, 2006). Furthermore, ingestion of amino acid supplements has been shown to enhance the fractional synthesis rate of protein in the soleus muscle (Carroll et al., 2005).

Leucine has been widely studied among all nutritional supplementations that were effective to prevent disuse muscle atrophy. It was documented that leucine, as an anabolic factor among all essential amino acids, has potential to affect muscle protein metabolism in several ways and is considered as a strong stimulator of protein synthesis (Katsanos et al., 2006). However, it is still unclear how leucine is sensed by the processes that regulate protein synthesis? Several *in vivo* and *in vitro* studies described that leucine plays an important role in improvement of protein synthesis via the IGF1-mTOR-Akt pathway (Kimball et al., 1999; Anthony et al., 2000b; Drummond et al., 2017). Anthony and co-workers reported that leucine is implicated in the stimulation of the eIF4E complex (that is, mRNA binding step in translation initiation; Anthony et al., 2000a). Branched-chain amino acids supplementation was observed to have an anabolic effect on human muscles during 14 days of bed rest, specifically, a slight improvement of protein synthesis in the early recovery period was observed (Stein et al., 1999, 2003). In addition, resistance exercise and essential amino acid supplementation were also shown to lead to preserve skeletal muscle mass and strength during 28-day bed rest (Brooks et al., 2008). Physical inactivity appears to inhibit mTORC1 signaling associated with reduced amino acid transporter protein contents thus suggesting that a blunted response in essential amino acid stimulation could be the underlying basis of muscle loss in older individuals (Hvid et al., 2010; Drummond et al., 2012). However, another study reported that supplementation of protein (0.8–1.5 g/kg/day) throughout 10 days of bed rest in older subjects promoted muscle strength but had no impact on muscle mass loss (Dillon et al., 2009). Discussions related to the efficiency of amino acid countermeasures in preventing protein mass losses during inactivity can be seen in the detailed review of Stein and associates (Stein and Blanc, 2011). In their conclusion, it is mentioned that various nutrition and protein supplements have different degrees of prevention and treatment of muscular atrophy, but the effect of these treatments is limited as a result of the blunted post absorptive and postprandial muscle protein synthesis after disuse atrophy. Therefore, searching for nutritional supplements with less synthetic metabolism resistance is undoubtedly an important research direction for the treatment of muscle atrophy.

## Future prospective

Over the past few decades, our current understanding of the cellular and molecular mechanisms involved in disuse muscle atrophy has significantly increased. However, this understanding remains incomplete with numerous unanswered questions. So far, the importance of protein turnover in driving disuse-induced muscle atrophy has been widely recognized. When the rate of protein synthesis becomes slower than the rate of protein breakdown, muscle atrophy begins. From a growing number of clinical and preclinical experiments, it is now clear that blunted or suppressed muscle protein synthesis seems to be the major drivers of disuse atrophy rather than increased muscle protein breakdown. However, further investigations are still needed to completely understand that how and why the activation of MuRF1/MAFbx, FOXO and other genes affect both protein synthesis and protein breakdown, which strongly suggests that increased proteolysis occurred somewhere. Additionally, it has been suggested frequently that during prolonged periods of disuse, oxidative stress can influence biochemical pathways and gene expression that regulates both muscle protein synthesis and breakdown. There are many factors leading to oxidative stress, such as low temperature, hypoxia, tissue damage, inflammation and calcium overload, etc., but which one among these factors contributing to oxidative stress in disuse-induced muscle atrophy need to be further elucidated.

On the other hand, to date, many studies have demonstrated beneficial effects of therapeutics countermeasure in disuse-induced muscle loss in model organisms. But we still lack of appropriate treatment strategies and have limited pharmaceutical options. The major hindrance, of course, is lack of knowledge regarding the cellular and molecular mechanisms involved in disuse muscle atrophy, which is far more complicated than we have been led to believe. There is much more to learn about how to prevent muscle atrophy as it is evidently not limited to muscle protein synthesis and breakdown only. Even as far as protein turnover is concerned, more accurate and targeted studies are needed to be done to unlock the secrets of abnormal or unbalanced protein metabolism underlying disuse muscle atrophy. More effective therapeutic agents or measures will emerge and develop along with the deepening of the research on the detailed mechanism in future.

## Author contributions

Conceived and designed by: YG and YA; Critical Analysis provided by: YG, YA, HW, and NG; Proof reading and edited the manuscript: YG, YA, HW, and NG; Wrote the paper: YG and YA; Figures drawn by: YA.

### Conflict of interest statement

The authors declare that the research was conducted in the absence of any commercial or financial relationships that could be construed as a potential conflict of interest.
